# Plant tolerance to excess light energy and photooxidative damage relies on plastoquinone biosynthesis

**DOI:** 10.1038/srep10919

**Published:** 2015-06-03

**Authors:** Brigitte Ksas, Noëlle Becuwe, Anne Chevalier, Michel Havaux

**Affiliations:** 1 CEA, IBEB, Laboratoire d’Ecophysiologie Moléculaire des Plantes, F-13108 Saint-Paul-lez-Durance, France; 2 CNRS, UMR 7265 Biologie Végétale et Microbiologie Environnementales, F-13108 Saint-Paul-lez-Durance, France; 3 Aix-Marseille Université, F-13284 Marseille, France

## Abstract

Plastoquinone-9 is known as a photosynthetic electron carrier to which has also been attributed a role in the regulation of gene expression and enzyme activities via its redox state. Here, we show that it acts also as an antioxidant in plant leaves, playing a central photoprotective role. When Arabidopsis plants were suddenly exposed to excess light energy, a rapid consumption of plastoquinone-9 occurred, followed by a progressive increase in concentration during the acclimation phase. By overexpressing the plastoquinone-9 biosynthesis gene *SPS1* (*SOLANESYL DIPHOSPHATE SYNTHASE 1)* in Arabidopsis, we succeeded in generating plants that specifically accumulate plastoquinone-9 and its derivative plastochromanol-8. The *SPS1*-overexpressing lines were much more resistant to photooxidative stress than the wild type, showing marked decreases in leaf bleaching, lipid peroxidation and PSII photoinhibition under excess light. Comparison of the *SPS1* overexpressors with other prenyl quinone mutants indicated that the enhanced phototolerance of the former plants is directly related to their increased capacities for plastoquinone-9 biosynthesis.

Photosynthesis inevitably produces toxic molecules derived from oxygen. Indeed, molecular oxygen can interact with the photosynthetic electron transport chain, leading to the formation of reduced forms of oxygen, such as superoxide, or with excited chlorophyll molecules, generating singlet oxygen (^1^O_2_)^1^,^2^,^3^. Superoxide can spontaneously or enzymatically dismutate into hydrogen peroxide which can subsequently lead to the hydroxyl radical in the presence of metals. Light-induced production of reactive oxygen species (ROS) is amplified under environmental stress conditions when the photosynthetic processes are inhibited and the absorption of light energy becomes excessive relative to the photosynthetic activity. One way to dissipate this excess energy is to transfer electrons and/or excitation to oxygen. However, to cope with the resulting production of harmful ROS, chloroplasts contain a variety of antioxidant mechanisms including soluble and lipophilic low molecular weight antioxidants^4^,^5^,^6^, detoxification enzymes and repair mechanisms^7^,^8^,^9^.

^1^O_2_ is produced within the photosystems (PS) from excited chlorophyll molecules in the triplet state^10^,^11^. ^1^O_2_ is thought to be the major ROS produced in plant cells at high light intensities^12^ and to be instrumental in the execution of ROS-induced cell death in leaves^13^. This ROS has a short lifetime (*ca*. 100 ns in biological tissues), suggesting a small diffusion path in cells^11^. Consequently, ^1^O_2_ reacts primarily in the close vicinity of its production site, and efficient ^1^O_2_ detoxification mechanisms must function close to the sites of ^1^O_2_ production. Accordingly, thylakoid membranes contain various lipid-soluble compounds that can quench ^1^O_2_ within the photosystems and around. Carotenoids are considered to be the first line of defense against ^1^O_2_ toxicity because of their high efficiency of ^1^O_2_ quenching and their localization in close proximity with the chlorophyll molecules in the light-harvesting complexes and the reaction centers of the photosystems^4^,^14^. However, prenyl lipids of the tocopherol family have also been shown to participate in the protection against ^1^O_2_. Tocopherols can quench ^1^O_2_, thus protecting PSII from photoinhibition, and can terminate lipid peroxidation chain-reactions, thus protecting the thylakoid membranes^6^,^15^,^16^,^17^,^18^. However, chloroplasts contain other prenyl lipids, such as plastoquinone-9, which could provide additional protection against photooxidative stress. Plastoquinones are viewed essentially as mobile electron carriers involved in electron transfer between PSII and PSI^19^. Through their redox state, there are also recognized as regulators of gene expression and enzyme activities^20^,^21^. However, it has been shown *in vitro* that plastoquinone-9 also has protective and antioxidant properties, being able to dissipate energy in the chlorophyll antennae^22^, to quench ^1^O_2_ and to inhibit oxidation of lipid membranes^23^,^24^,^25^. If this function does occur *in vivo*, it could be of great physiological importance because plastoquinones are diffusible molecules present in relatively high amounts in the thylakoids (estimated to be ~7 molecules per PSII reaction center, ref. 26). Moreover, both the head group and the isoprenoid side chain of plastoquinol are able to quench ^1^O_2_, likely making this molecule a better antioxidant than tocopherols. The possible role of plastoquinone-9 *in planta* as an antioxidant and photoprotector is analyzed here in leaves of the model plant *Arabidopsis thaliana*. To this end, the plastoquinone biosynthesis pathway was manipulated to generate plants that contain noticeably more plastoquinone-9 than the wild type (WT), and the behavior of those plastoquinone-accumulating plants under high light stress conditions was compared to that of wild-type (WT) plants.

## Results

### The plastoquinone-9 content of Arabidopsis leaves is highly sensitive to excess light energy

**Figure 1:**
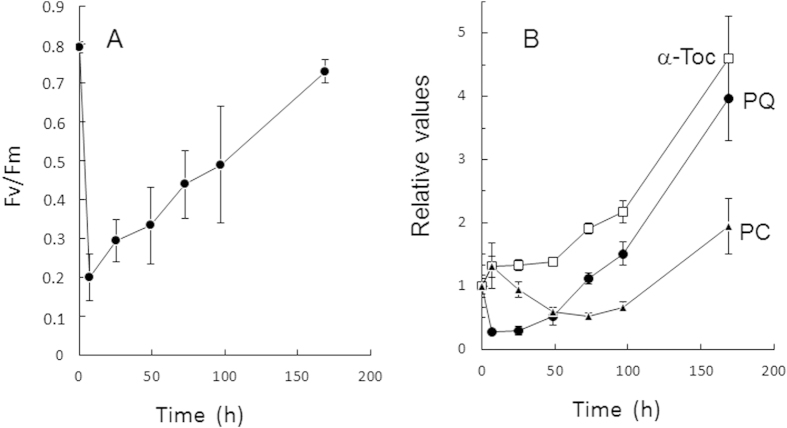
Responses of WT Arabidopsis plants to excess light energy. Plants (Col 0) aged 4 weeks were grown at a PFD of 170 μmol photons m^−2^ s^−1^. Plants were exposed to high light (PFD, 1300 μmol photons m^−2^ s^−1^) and low air temperature (5 °C). **A**) Maximal quantum yield of PSII photochemistry (Fv/Fm chlorophyll fluorescence ratio). Data are mean values of 10 measurements + SD. **B**) Changes in prenyl lipid concentrations in relative values: α-tocopherol (α-Toc), plastochromanol-8 (PC) and total plastoquinone-9 (PQ). For each compound, values were normalized to the control value at time 0. Data are mean values of 4 measurements + SD. A value of 1 corresponds to 1.84 nmol cm^−2^ for plastoquinone-9, 19.3 pmol cm^−2^ for plastochromanol-8 and 270 ng cm^−2^ for α-tocopherol.

High excitation pressure on the photosystems can be achieved by increasing light irradiance and/or decreasing temperature^27^. WT Arabidopsis plants aged 4 weeks were suddenly exposed to high photon flux density (PFD, 1300 μmol photons m^−2^ s^−1^) and low air temperature (5 °C). The decreased air temperature led to leaf temperature (around 18 °C) in the temperature range of control leaves grown at 170 μmol photons m^−2^ s^−1^, thus avoiding leaf heating usually associated with high PFDs. Under those conditions, PSII underwent rapid photoinhibition, as shown by the fall of the Fv/Fm chlorophyll florescence ratio from 0.8 to 0.2 after 7 h in high light (Fig. 1A). Then, PSII slowly recovered, with Fv/Fm progressively increasing and finally reaching, after 5 d in high light, values close to the initial values recorded before stress. Plastoquinone-9 (reduced + oxidized) appeared to follow the same trends: it drastically decreased by 80% after 7 h in high light (Fig. 1B). Subsequently, the total plastoquinone-9 concentration increased, finally leading to a strong accumulation corresponding to almost 4 times the plastoquinone-9 levels measured before stress. Those changes in plastoquinone-9 concentration were associated with limited changes in the plastoquinone reduction state: the plastoquinone-9 reduction levels decreased from ca. 80% reduction to ca. 70% during the high light treatment and increased back to the initial values after 5 d in high light (Supplementary Fig. 1). The plastochromanol-8 content also changed with the light conditions, but compared to plastoquinone-9, the changes were very attenuated and occurred much more slowly (Fig. 1B). In contrast with plastochromanol-8 and plastoquinone-9, no loss of α-tocopherol was observed during high light stress: the α-tocopherol concentration remained stable during the first two days of light stress and then it increased noticeably.

**Figure 2:**
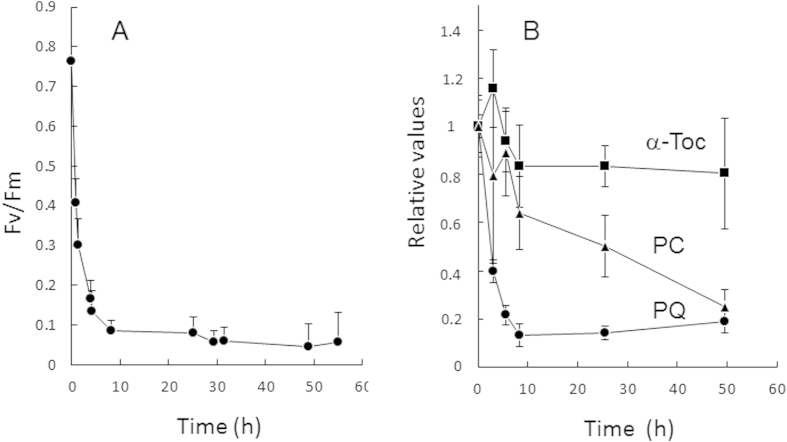
Responses of the Arabidopsis ch1 mutant to excess light energy. Plants grown for 5 weeks at PFD 170 μmol photons m^−2^ s^−1^ were exposed to high light (PFD, 1000 μmol photons m^−2^ s^−1^) and low temperature (10 °C). **A**) Maximal quantum yield of PSII photochemistry (Fv/Fm chlorophyll fluorescence ratio). Data are mean values of 10 measurements + SD. **B**) Changes in prenyl lipid concentrations in relative values: α-tocopherol (α-Toc), plastochromanol-8 (PC) and total plastoquinone-9 (PQ). For each compound, values were normalized to the control value at time 0. Data are mean values of 4 measurements + SD. A value of 1 corresponds to 1.9 nmol cm^−2^ for plastoquinone-9, 16.8 pmol cm^−2^ for plastochromanol-8 and 539 ng cm^−2^ for α-tocopherol.

We also analyzed a photosensitive Arabidopsis mutant, the *ch1* mutant, which has been shown to release more singlet oxygen (^1^O_2_) from the PSII reaction centers compared to WT^28^. Because of their high photosensitivity, *ch1* mutant plants aged 5 weeks were exposed to less severe stress conditions than those used for WT in Fig. 1: 1000 μmol photons m^–2^ s^–1^ at ^1^0°C. Nevertheless, this milder treatment caused a drastic inhibition of PSII photochemistry which did not reverse (Fig. 2A). Again, plastoquinone-9 underwent drastic changes in concentration during the high light treatment which mirrored the inhibition of PSII photochemistry (Fig. 2B). There was a decrease in the percentage reduction state of plastoquinone-9 from *ca*. 80% at time 0 to *ca*. 60% at the end of the experiments after 50 h of light stress (Supplementary Fig. 1). Plastochromanol-8 behaved similarly to plastoquinone-9, exhibiting a noticeable decrease, but this effect was delayed relative to the plastoquinone-9 changes. In comparison, α-tocopherol exhibited very limited changes and remained close to the control levels before stress. To sum up, among the prenyl lipids of Arabidopsis leaves, plastoquinone-9 appeared to be by far the most sensitive to the light conditions, displaying strong reductions during photooxidative stress and marked accumulations during stress acclimation. In addition, we observed a correlation between the changes in PSII activity and the changes in plastoquinone-9 concentration.

### High light-induced changes in the expression of genes involved in the plastoquinone-9 biosynthesis pathway

**Figure 3:**
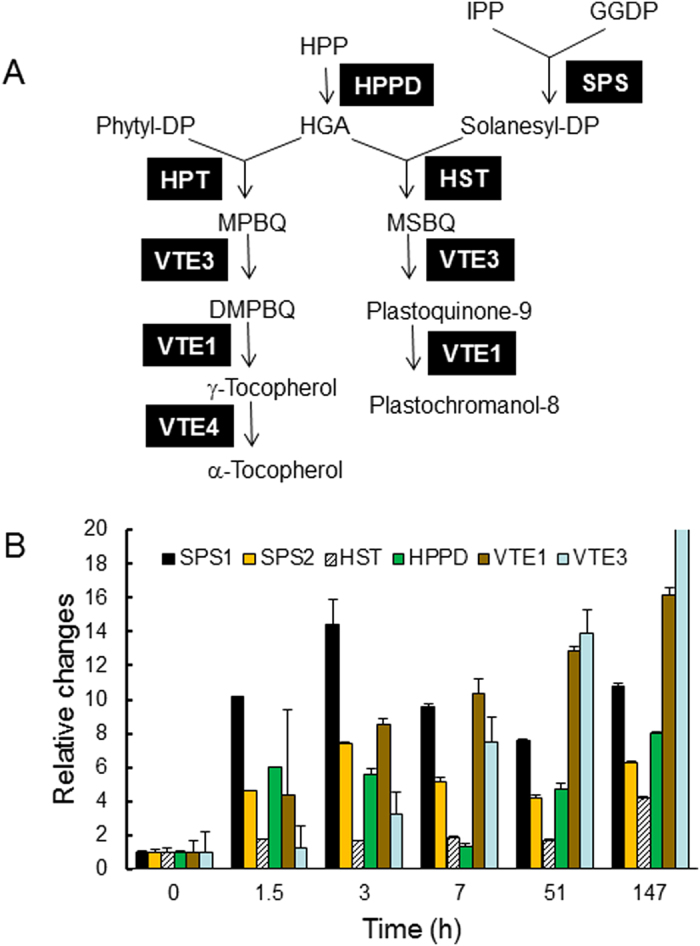
Light regulation of the plastoquinone-9 biosynthesis pathway. **A**) Scheme of the biosynthesis pathway. **B**) qRT-PCR measurements of the expression of several key genes involved in the biosynthesis of plastoquinone-9 in leaves of WT Arabidopsis plants exposed to high light (1300 μmol photons m^−2^ s^−1^ at air temperature of 7 °C). Data are normalized to the values at time 0. Data are mean values of 3 separate experiments +SD. The expression level of *VTE3* at time 147 h (87 + 2) is out of scale in the graph. Metabolites: HPP, hydroxyphenylpyruvate; HGA, homogentisate; DP, diphosphate; IPP, isopentenyl phosphate; GGDP, geranylgeranyl diphosphate; MPBQ, methy-phytyl-benzoquinone; MSBQ, methyl-solanesyl-benzoquinone, DMPBQ, dimethyl-phytyl-benzoquinone. Enzymes: HPPD, HPP dioxygenase; HST, homogentisate solanesyl diphosphate transferase; SPS, solanesyl disphosphate synthase; VTE1, tocopherol cyclase; VTE2, homogentisate phytyl transferase; VTE3, MPBQ/MSBQ methyl transferase; VTE4, γ-tocopherol methyl transferase.

A scheme of the biosynthetic pathways of plastoquinone-9, plastochromanol-8 and α-tocopherol is presented in Fig. 3A. The first committed step in plastoquinone-9 biosynthesis is the condensation of the aromatic compound homogentisate (HGA) with solanesyl diphosphate by homogentisate solanesyl diphosphate transferase (HST), leading to the formation of MSBQ (methyl-solanesyl-benzoquinone). HGA is shared in common with the tocopherol biosynthetic pathway, with prenylation of HGA with phytyl diphosphate producing MPBQ (methyl-phytyl-benzoquinone), a precursor of α-tocopherol. MPBQ is converted to α-tocopherol by the action of VTE3, a methyl transferase^29^ that is also involved in plastoquinone-9 biosynthesis by converting MSBQ (methyl-solanesyl-benzoquinone) to plastoquinone.

HGA synthesis from hydroxyphenylpyruvate (HPP) is catalyzed by HPP dioxygenase (HPPD) while solanesyl diphosphate is synthesized from geranylgeranyl diphosphate (GGDP) and isopentenyl phosphate (IPP) by a reaction catalyzed by solanesyl diphosphate synthase (SPS). Three SPS enzymes are present in Arabidopsis, SPS1, SPS2 and SPS3^30^,^31^. While SPS3 is a ubiquinone biosynthetic enzyme localized in mitochondria^31^, SPS1 and SPS2 have been recently demonstrated to be targeted to plastids and to be involved in plastoquinone biosynthesis^32^. Previously, SPS1 was supposed to be involved in the synthesis of the side chain of ubiquinone while plastoquinone biosynthesis was believed to be dependent on SPS2 only^33^. However, this view has been challenged by recent observations revealing that a mitochondria-targeted gene (*SPS3*) different from *SPS1* and *SPS2* is the main, if not sole, contributor of solanesyl diphosphate synthase activity required for ubiquinone biosynthesis^31^. Moreover, the Arabidopsis mutants *AtSPS1* and *AtSPS2* were found to be affected in plastoquinone-9 and plastochromanol-8 biosynthesis, not in ubiquinone-9 synthesis. Thus, the current view is that both SPS1 and SPS2 catalyze the elongation of the prenyl side chain of plastoquinone^32^. Plastochromanol-8 has been demonstrated to originate from reduced plastoquinone-9 through the action of VTE1^34^,^35^, a tocopherol cyclase enzyme also involved in the biosynthesis of α-tocopherol from its direct precursor, γ-tocopherol (Fig. 3A).

In Fig. 3B, we examined the effect of excess light energy on the expression of several genes of the plastoquinone-9 and plastochromanol-8 biosynthesis pathway. One can see that the expression of both *SPS1* and *SPS2* genes was rapidly induced after transfer of plants aged 4 weeks from low light to high light, with the accumulation of *SPS1* transcripts being noticeably more pronounced than that of *SPS2*. The expression pattern of *HPPD* was close to that of *SPS2*, with an induction in high light. In striking contrast, *HST* expression was not affected by light. The *VTE1* and *VTE3* genes were also activated by high light but this effect was more progressive and continuous than the up-regulation of *SPS1*, *SPS2* and *HPPD*. So, the plastoquinone-9 biosynthesis pathway is globally up-regulated by high light, with a marked effect on the *SPS1* gene in less than 3 h after the transfer from low light to high light. Light induction of the plastoquinone pathway is consistent with early data on the incorporation of radiolabelled tyrosine into prenyl lipids^36^. Upon illumination of *Xanthium* leaves, incorporation of radioactivity into plastoquinone was observed to be much more pronounced and to occur more rapidly than incorporation into tocopherols.

### Synthesis of plastoquinone-9 and plastochromanol-8 is boosted in SPS1-overexpressing plants and correlates with tolerance to excess light energy

**Figure 4:**
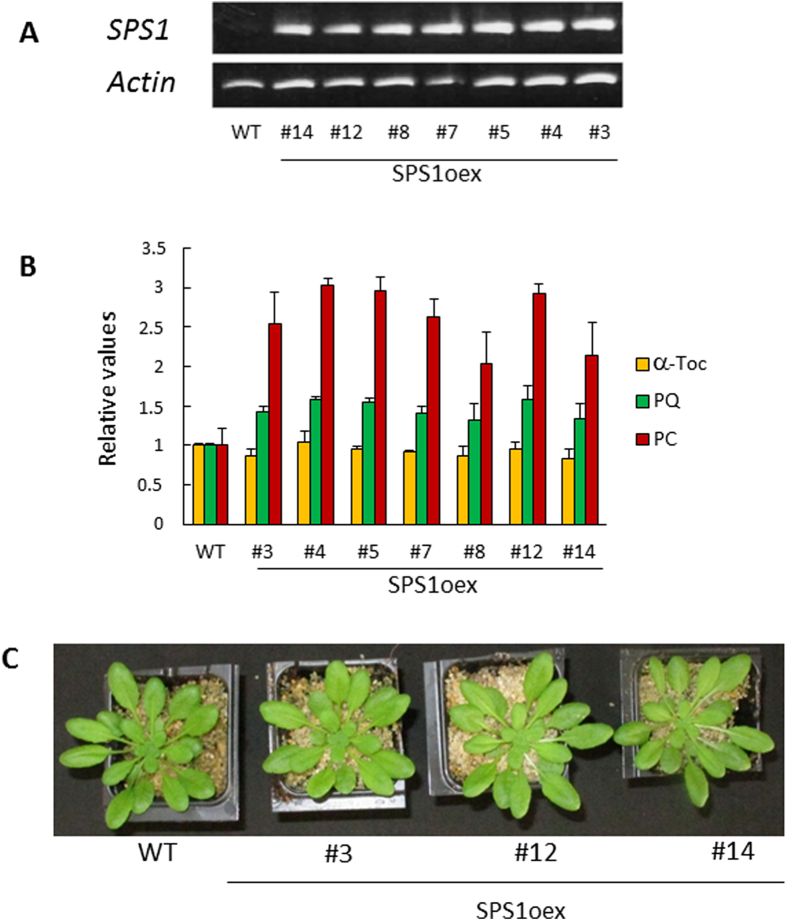
Overexpression of the *SPS1* gene in Arabidopsis. A) RT-PCR analysis of SPS1 transcripts in WT (col 0) and SPS1oex transformed lines. *ACTIN2* mRNAs were used as loading controls. The *SPS1* and *ACTIN2* images are from the same experiment and are therefore directly comparable. **B**) Plastoquinone-9 (PQ), plastochromanol-8 (PC) and α-tocopherol (α-Toc) levels in leaves. Values (measured on a leaf area basis) are normalized to the WT values. Data are mean values of 4 separate experiments + SD. The leaf specific weight (in mg dry weight cm^−2^) did not differ significantly between the genotypes: 1.1460 + 0.1850 for Col 0, 1.2203 + 0.0808 for SPS1oex #3, 1.1670 + 0.0813 for SPS1oex #12 and 1.1354 + 0.1485 for SPS1oex #14. **C**) Growth phenotype of the plants (WT and 3 SPS1oex lines). Plants were grown for 4 weeks at PFD 170 μmol m^−2^ s^−1^.

**Figure 5:**
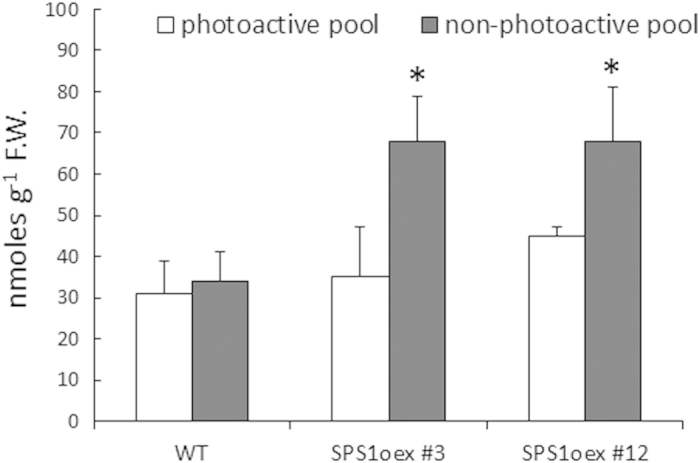
Pool size of photoactive and non-photoactive plastoquinone-9. Measurements were performed as detailed in the Methods section on leaves of WT Arabidopsis and two SPS1oex lines (#3 and #12). Data are mean values of 3 separate experiments + SD. F.W. = fresh weight. The stars indicate significant difference between WT and the SPS1oex lines at P < 0.05 (Student’s t test).

A previous work has shown that constitutive overexpression of *HST* in Arabidopsis has little effect on the plastoquinone-9 concentration in leaves^37^, suggesting that HST activity is not the limiting step for plastoquinone-9 biosynthesis. Our observation that *HST* gene expression is not responsive to a condition associated with plastoquinone-9 accumulation could be seen as a fact in line with this suggestion. Considering the strong and rapid expression of *SPS1* under conditions that induced plastoquinone-9 accumulation in leaves (Figs. 1,3), we decided to overexpress this gene in Arabidopsis. Arabidopsis *SPS1* cDNA was inserted under the control of the 35S promoter in a plant binary vector. This vector was used to generate transgenic Arabidopsis plants, and a number of stable lines derived from independent transformation events were obtained. Figure 4A shows a selection of homozygous lines (SPS1oex) exhibiting a strong accumulation of *SPS1* transcripts. This transformation had marked effects on plastoquinone-9 and its derivative plastochromanol-8 which accumulated in all lines (Fig. 4B). The effect was particularly marked for plastochromanol-8 with an accumulation factor of 2 to 3. The plastoquinone-9 accumulation was less marked, but nevertheless reached approximately 150-170% of the WT level. The reduction state of plastoquinone-9 increased very slightly in the SPS1oex leaves (Supplementary Fig. 2). In contrast, the α-tocopherol concentration did not change significantly in any of the transformed lines. Similarly, the levels of minor forms of tocopherol (δ- and γ-tocopherol) were not modified by the SPS1 overexpression (data not shown). Thus, constitutive overexpression of *SPS1* selectively boosted the plastoquinone/plastochromanol pathway, as expected. Importantly, plastoquinone-9 and plastochromanol-8 accumulation in SPS1oex plants had no effect on growth (Supplementary Fig. 3A), did not modify the quantum yield of photosynthetic electron transport (Supplementary Fig. 3B) and did not affect the chlorophyll levels (Supplementary Fig. 3C). This could suggest that the extra plastoquinone-9 molecules that accumulate in the SPS1oex lines are not stored in the thylakoid membranes and are not connected to the photosynthetic electron transport chain. The pool of photoactive plastoquinone-9 (connected to the electron transport chain) was estimated in Fig. 5. The size of this pool did not differ significantly between SPS1oex leaves and WT leaves. In contrast, the pool of non-active plastoquinones was noticeably enlarged in the *SPS1*-overexpressing leaves, suggesting plastoquinone-9 storage in a compartment different from the thylakoid membranes.

**Figure 6:**
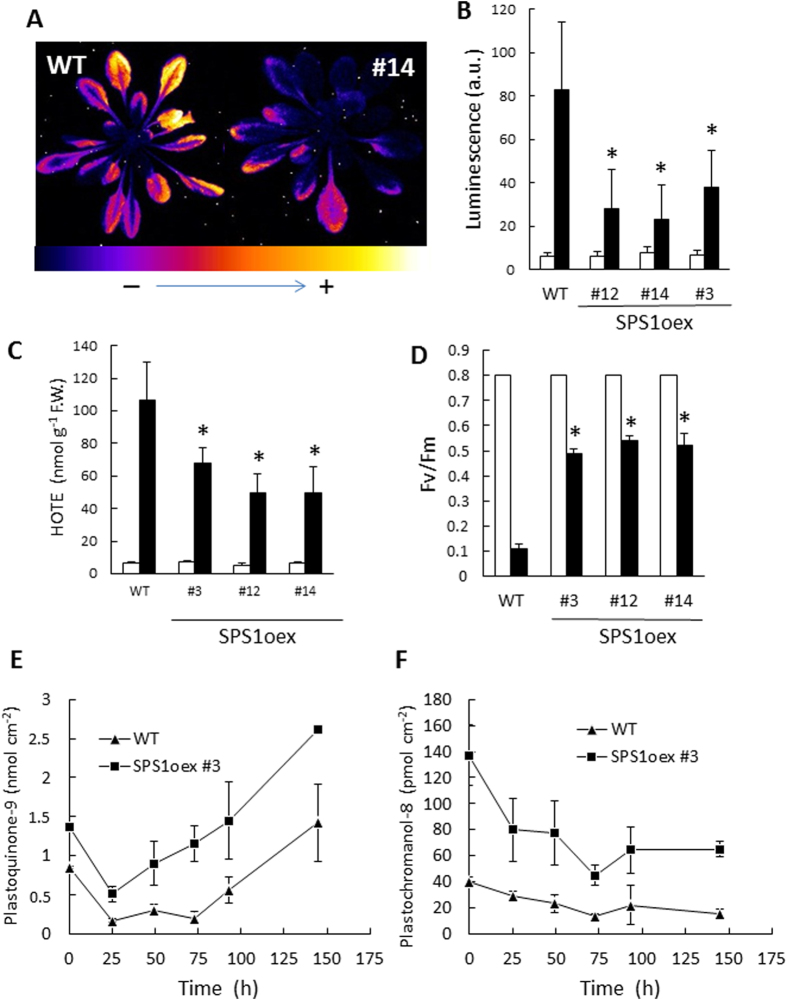
Tolerance of *SPS1*-overexpressing Arabidopsis plants to high light stress. Plants grown for 5 weeks at a PFD of 110 μmol photons m^−2^ s^−1^ were exposed to high light at low temperature (1300 μmol photons m^−2^ s^−1^ and 5 °C) for 28 h. **A**) Lipid peroxidation monitored by autoluminescence imaging in WT (col 0) and SPS1oex #14 Arabidopsis plants after the light stress. **B**) Average autoluminescence intensity in WT and several SPS1oex lines before and after high light stress (white and black bars, respectively). Data are mean values of 5 measurements + SD. **C**) HOTE concentrations before and after high light stress (white and black bars, respectively). Data are mean values of 3 measurements + SD. **D**) Maximal quantum yield of PSII photochemistry (Fv/Fm) after high light stress before and after high light stress (white and black bars, respectively). Data are mean values of 10 measurements + SD. **E-F**) Changes in plastoquinone-9 (panel E) and plastochromanol-8 (panel F) in WT and the SPS1oex #3 line during high light stress. Data are mean values of 4 measurements + SD. In panels B, C and D, the stars indicate significant difference between WT and the SPS1oex lines at P < 0.05 (Student’s t-test).

The plastoquinone/plastochromanol-accumulating SPS1oex plants were exposed to photooxidative stress. We chose rather severe stress conditions in order to induce photooxidative damage in WT plants: 1300 μmol photons m^−2^ s^−1^ at 5 °C with a photoperiod of 13 h. Moreover, for this experiment, plants were grown at the PFD of 110 μmol m^−2^ s^−1^ to increase the sensitivity of WT plants to high light stress. As shown by the autoluminescence image of Fig. 6A and the quantification of the autoluminescence signal in Fig. 6B, WT accumulated lipid peroxides after 28 h of such stress treatment. The increased oxidation of lipids was confirmed by the amount of hydroxy octadecatrienoic acid (HOTE, the hydroxy fatty acid derived from the oxidation of linolenic acid, the most abundant fatty acid in Arabidopsis leaves), which drastically increased in WT leaves (Fig. 6C). In striking contrast, the SPS1oex lines were much less sensitive to the light stress, with a noticeable reduction of lipid peroxidation (Fig. 6A-C). Extensive leaf bleaching was observed in WT plants, but not in the SPS1oex lines (data not shown). The increased phototolerance of SPS1oex lines is confirmed by the maintenance of higher PSII photochemical efficiency (Fv/Fm) compared to WT (Fig. 6D).

Figures 6E and 6F show the time course of the changes in total plastoquinone-9 and in plastochromanol-8 in one of the SPS1oex lines (#3) in high light. Plastoquinone-9 initially decreased in high light-stressed WT and SPS1oex plants. Subsequently, the plastoquinone concentration increased, as previously shown in Fig. 1, but this effect was much more rapid and more pronounced in the SPS1oex line compared to WT. Thus, compared to WT, the plastoquinone-9 levels were much higher in the *SPS1* overexpressors throughout the stress treatment, and the difference was particularly marked during the acclimation phase after several days in high light. After 6 d in high light, the plastoquinone-9 levels were almost doubled in the SPS1oex line relative to WT. The plastoquinone reduction state was slightly higher in the SPS1oex #3 line relative to WT, but it did not change much with the light stress conditions (Supplementary Fig. 2). In contrast with plastoquinone-9, plastochromanol-8 was observed to decrease in both types of plants during the stress experiment (Fig. 6F).

### The phototolerance of tocopherol-deficient Arabidopsis *vte* mutants

**Figure 7:**
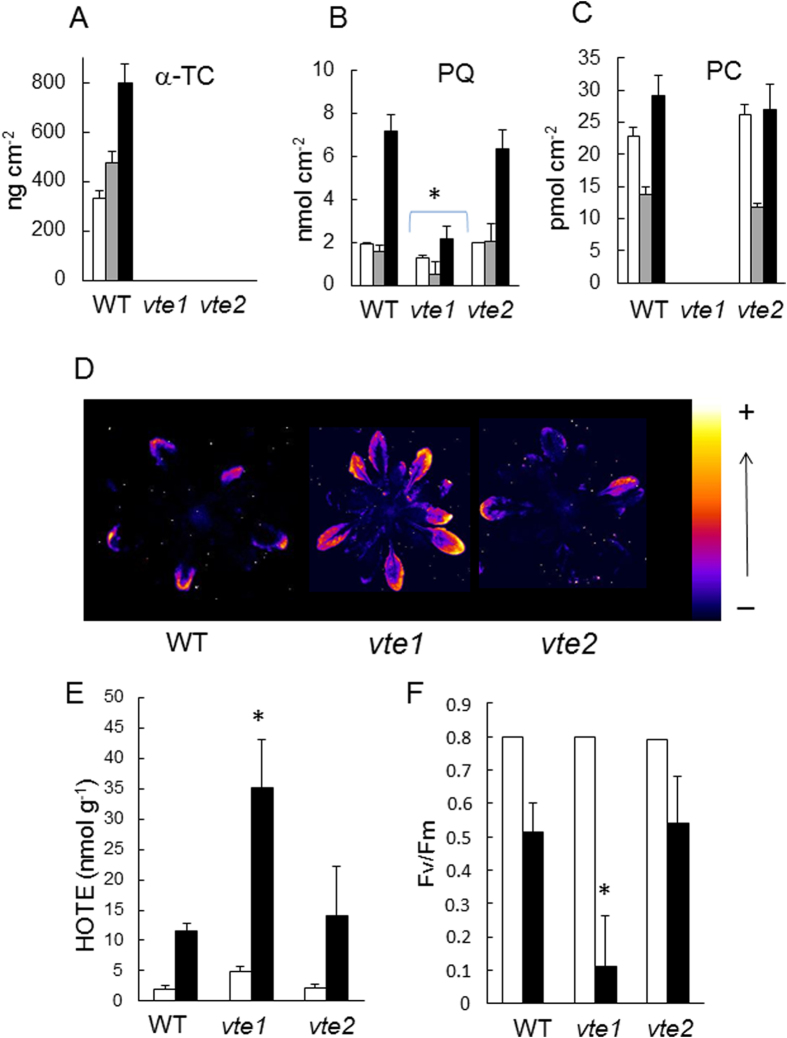
Comparison of the vte1 and vte2 mutants exposed to excess light energy. WT (Col 2) and mutant plants were grown for 4 weeks at a PFD of 170−μmol photons m^−2^ s^−1^. Plants were then exposed to high light (1300 μmol m^−2^ s^−1^ at 8 °C). **A–C**) Prenyl lipid levels (α-tocopherol [α-Toc], total plastoquinone-9 [PQ], plastochromanol-8 [PC]) before and after exposure for 3 or 7 d to high light at low temperature (white, grey and black bars, respectively). Data are mean values of 4 experiments + SD. Leaf specific weight (in mg dry weight cm^−2^) did not differ significantly between the genotypes so that expression of the data on a leaf area basis or on a weight basis does not affect the conclusions: 1.467 + 0.075 for WT, 1.470 + 0.044 for *vte1* and 1.420 + 0.081 for *vte2*. **D**) Lipid peroxidation monitored by autoluminescence imaging after 28 h in high light. **E**) HOTE concentrations before and after high light stress (white and black bars, respectively). Data are mean values of 3 measurements + SD. **F**) PSII photochemical efficiency (Fv/Fm chlorophyll fluorescence ratio) measured before and after high light stress (white and black bars, respectively). Data are mean values of 12 measurements + SD. Panels B, E, F: the stars indicate significant difference between WT and the mutants at P < 0.05 (Student’s t-test).

The *vte1* and *vte2* mutants are deficient in tocopherol cyclase and homogentisate phytyl transferase, respectively, leading to a complete deficiency in tocopherols^38^,^39^, as confirmed in Fig. 7A. As previously reported^34^,^35^, *vte1* is also deficient in plastochromanol-8 while the plastochromanol-8 content of *vte2* leaves is normal (Fig. 7C). Interestingly, the combined lack of tocopherols and plastochromanol-8 in *vte1* leaves was associated in a substantial decrease in plastoquinone-9 levels (Fig. 7B). Under normal growth conditions, the plastoquinone-9 pool was decreased to less than 75% of the WT level. As previously shown^40^,^41^, neither chlorophylls nor carotenoids are affected in the *vte1 and vte2* mutants. Under high light stress, the plastoquinone-9 concentration fell to less than 50% of the WT level. In contrast, loss of tocopherol with normal levels of plastochromanol-8 in the *vte2* mutant had no impact on the plastoquinone-9 concentration. When exposed to high light stress (1300 μmol m^−2^ s^−1^ and 8 °C), pronounced photooxidative stress occurred in *vte1* leaves, as illustrated by the accumulation of lipid peroxides (Fig. 7D), the increase in hydroxy fatty acids (HOTEs) (Fig. 7E), the extensive leaf bleaching (data not shown), and the loss of PSII activity (Fv/Fm) (Fig. 7F). These effects were attenuated in WT and *vte2* plants which behaved similarly in response to high light stress. One can thus conclude that loss of tocopherol has rather limited effects on the photosensitivity of Arabidopsis, unless plastochromanol-8 is also missing. This suggests a functional overlap between tocopherols and plastochromanol-8 under high light stress. Our results also show that the protective action of those compounds is required to preserve the plastoquinone pool size.

### The phototolerance of VTE1-overexpressing plants

**Figure 8:**
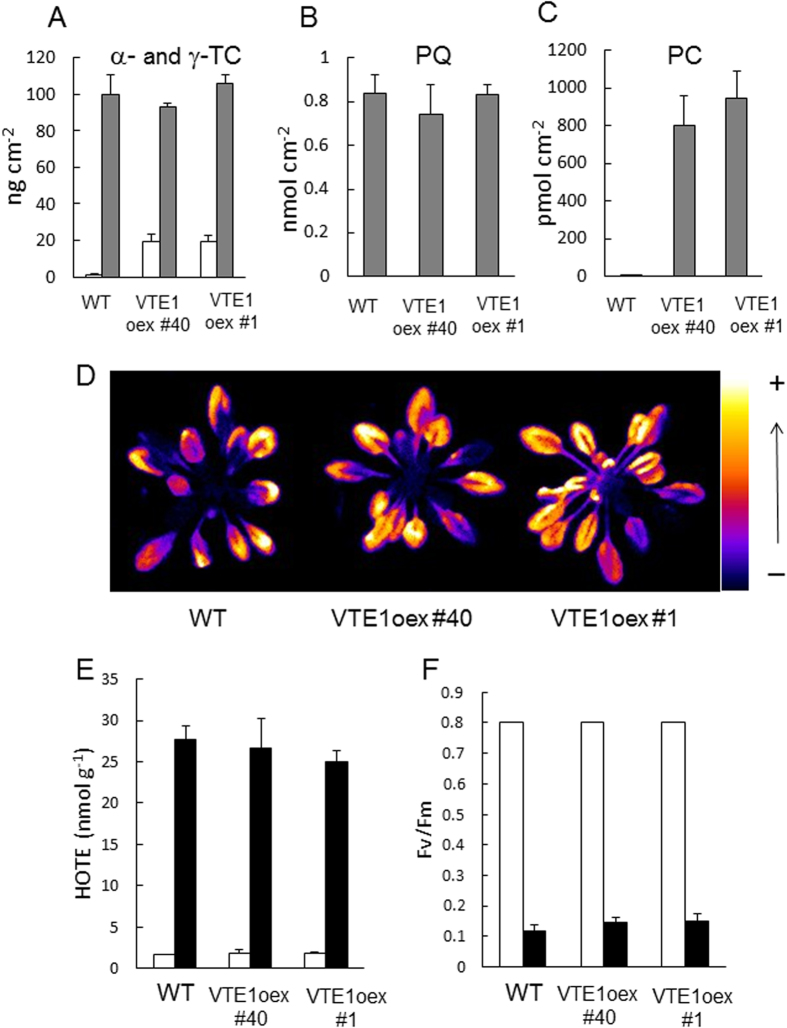
Responses of *VTE1*-overexpressing Arabidopsis plants to excess light energy. WT (Col 2) and two VTE1oex lines (#1 and 40) were were grown for 5 weeks at a PFD of 110 μmol photons m^−2^ s^−1^. Plants were then exposed to high light (1300 μmol m^−2^ s^−1^ at 5 °C). A-C) Prenyl lipid levels under control conditions (before light stress): **A**) γ-tocopherol (γ-Toc, white bars) and α-tocopherol (α-Toc, grey bars); **B**) plastoquinone-9 (PQ); **C**) plastochromanol-8 (PC). Data are mean values of 4 measurements + SD. The leaf specific weight (mg dry weight/cm^2^) did not differ significantly between the genotypes: 1.1601 + 0.0980 for Col 2, 1.1673 + 0.1319 for VTE1oex #40 and 1.1673 + 0.1491 for VTE1oex #1. **D**) Lipid peroxidation monitored by autoluminescence imaging after 28 h in high light. **E**) HOTE concentrations before and after high light stress (white and black bars, respectively). Data are mean values of 3 measurements + SD. **F**) PSII photochemical efficiency (Fv/Fm chlorophyll fluorescence ratio) before and after high light stress (white and black bars, respectively). Data are mean values of 12 measurements + SD.

Overexpression of the *VTE1* gene in Arabidopsis has two effects: it brings about a strong accumulation of plastochromanol-8^35^ and it enhances the production of γ-tocopherol^40^, as expected from the functions of this gene in the biosynthesis pathway (Fig. 3A) and as confirmed in Fig. 8A–C. The plastoquinone-9 levels were not significantly affected by the genetic modification (Fig. 8B). Also, the concentration of α-tocopherol was not modified in the VTE1 overexpressor (Fig. 8A), so that the increase in the total amount of tocopherols (α + γ) was rather moderate (ca. 120 vs 100 ng cm^−2^). γ-Tocopherol is a potent antioxidant *in vivo*, reducing oxidative stress-induced lipid peroxidation even better than α-tocopherol^42^. In some plant species such as runner bean, young leaves contain very high levels of γ-tocopherol, supplementing other lipophilic antioxidants and contributing to the overall protection against oxidative stress^43^. So, the phototolerance of the VTE1 overexpressors should not be negatively affected by the changes in tocopherol composition. Neither the growth rate, nor the chlorophyll content of the VTE1 overexpressors was different from WT values (total chlorophyll in μg cm^−2^: 23.51 + 1.85 for WT, 23.98 + 1.06 for VTE1oex #1 and 24.12 + 0.64 for VTE1oex#40). Both WT and the VTE1 overexpressors were exposed to a high light stress condition that induced photodamage and lipid peroxidation in WT leaves. To this end, we decreased the growth PFD (to 110 μmol m^−2^ s^−1^) and increased the severity of the stress treatment (as in Fig. 6). Under those conditions, WT plants exhibited high autoluminescence emission, high HOTE levels and low Fv/Fm ratios. The VTE1oex lines behaved like WT: pronounced lipid peroxidation (Fig. 8D-E) and extensive PSII photoinhibition (Fig. 8F) were observed in all plants. Thus, accumulation of plastochromanol-8 itself is not sufficient to provide significant protection against photostress. This observation is in line with previous data obtained by Zbierzak *et al*.^35^ who did not find any change in photosynthesis in the VTE1oex lines after several days in high light.

## Discussion

Plants are known to accumulate tocopherols in response to high PFDs (e.g.^15^,^41^,^44^,^45^). In comparison, the light dependence of plastoquinone-9 and plastochromanol-8 levels is poorly documented. A few reports indicated that plastoquinone-9 accumulates in leaves exposed to high PFDs^34^,^46^,^47^,^48^. In contrast, the changes in plastochromanol-8 content of Arabidopsis leaves induced by high light stress were observed to be small compared to plastoquinone-9^32^,^34^,^35^,^49^. The present study shows that the plastoquinone-9 concentration in Arabidopsis leaves is extremely sensitive to the light conditions. Exposure to excess light energy by transferring low light-grown Arabidopsis plants to high light induced a dramatic reduction of the total plastoquinone-9 content while longer-term acclimation to high light was associated with a marked rise in the plastoquinone-9 levels (Fig. 1B). Moreover, those changes in plastoquinone-9 content appeared to follow the changes in PSII photochemical efficiency, with PSII photoinhibition being accompanied by a loss of plastoquinone-9 and PSII recovery being paralleled by increasing concentrations of plastoquinone-9. This correlation with PSII activity was not found for plastochromanol-8 and α-tocopherol. Although the changes in plastoquinone-9 and plastochromanol-8 concentrations induced by high light followed the same trends, the plastochromanol changes were delayed and occurred with much lower amplitude relative to the plastoquinone changes (Fig. 1B). After transfer to high PFDs, the tocopherol content remained constant while PSII photochemistry was strongly inhibited. These changes were amplified in the ^1^O_2_-overproducing *ch1* Arabidopsis mutant (Fig. 2), confirming the close correlation between PSII photochemical efficiency and plastoquinone-9 content under high light stress as well as the temporal disconnection between PSII photoinhibition and the variations in plastochromanol-8 and tocopherol contents.

PSII photoinhibition is related to ^1^O_2_ formation resulting from the interaction between molecular oxygen and the triplet excited state of the reaction center chlorophyll molecule P680^10^ and is linked to the degradation of the D1 protein triggered by the formed ^1^O_2_^50^,^51^. The involvement of ^1^O_2_ in the damage of the PSII reaction centers can also be indirect by inhibiting synthesis of D1 and impairing the repair processes^52^. Elimination of ^1^O_2_ produced in the PSII centers is believed to be fulfilled by the β-carotene molecules located in the PSII reaction center^53^ as well as by α-tocopherol^16^,^54^,^55^. However, plastoquinone-9 has been demonstrated to be another potent antioxidant, which is able to quench ^1^O_2_ *in vitro*^23^,^25^,^56^ and to inhibit lipid peroxidation in model systems^24^. Addition of plastoquinone homologues to Chlamydomonas cultures grown in the presence of plastoquinone biosynthesis inhibitors prevented degradation of D1^55^. Moreover, plastoquinone-9 and plastochromanol-8 incorporated into liposomes were found to be more active than α-tocopherol in the inhibition of ^1^O_2_-induced lipid peroxidation^24^, and plastoquinone-9 was observed to be a better quencher of ^1^O_2_ than α-tocopherol in solvents^23^. Thus, the dramatic loss of plastoquinone-9 observed in our study in Arabidopsis leaves suddenly exposed to excess light energy (Fig. 1B) is likely to be related to the ^1^O_2_-scavenging activity of this compound which involves oxidation and consumption of the prenyl-lipidic molecule. Accordingly, increased production of ^1^O_2_ from the PSII centers in the *ch1* mutant was associated with an accelerated loss of plastoquinone-9. It is clear that exhaustion of the available pool of plastoquinones under excess light energy can have important consequences for the PSII repair cycle by precluding PSII reassembly. This phenomenon can exacerbate the inhibition of PSII photochemical activity. However, as shown here, exposure of Arabidopsis plants to high PFDs triggered up-regulation of the plastoquinone biosynthesis pathway, thus enhancing the capacity for plastoquinone-9 synthesis during plant acclimation to high light and compensating for the initial loss of plastoquinone-9 (Fig. 3B). This phenomenon led *in fine* to a strong accumulation of plastoquinone-9 in photoacclimated Arabidopsis plants.

Importantly, the present study shows that constitutive enhancement of the plastoquinone-9 biosynthesis capacity of Arabidopsis by overexpressing the *SPS1* gene strongly reinforces the tolerance of Arabidopsis plants to photooxidative stress (Fig. 6). The SPS1oex transgenic plants contain more plastoquinone-9 than WT plants both in low light and in high light conditions. Particularly, the high light stress-induced increase in plastoquinone-9 concentration following the initial reduction was much more rapid and reached much higher levels in SPS1oex plants compared to WT plants (Fig. 6E). Also, the plastoquinone-9 depletion in leaves immediately after transfer to high light was less pronounced in the SPS1oex plants relative to WT, leading to plastoquinone-9 contents close to the control values measured in WT plants before stress. The enhanced plastoquinone-9 levels in the SPS1oex lines throughout the high light stress treatments compared to WT were accompanied by a marked decrease in photooxidative damage and lipid peroxidation and in the preservation of the PSII photochemical activity. These findings show that active and efficient synthesis of plastoquinone-9 plays a role in the photoprotection mechanisms of plants. Incidentally, our results also suggest that solanesyl diphosphate synthesis is a limiting step in the plastoquinone biosynthesis pathway.

It should be stressed that, in addition to plastoquinone-9 accumulation, plastochromanol-8 levels were also noticeably increased in the SPS1oex lines. Plastochromanol-8 is synthesized from reduced plastoquinone-9 by VTE1^34^,^35^. Thus, the strong accumulation of plastochromanol-8 in the SPS1oex lines suggests that chloroplasts can accommodate a finite amount of plastoquinone molecules, with the excess molecules being converted to plastochromanol-8. The structure of plastochromanol-8 is similar to that of tocotrienols and it has been shown *in vitro* that, like tocotrienols, it has antioxidant properties^23^,^57^. Plastochromanol-8 has also been reported to play a crucial role in lipid protection in Arabidopsis seeds in combination with tocopherols^58^. Although the protective role of this compound in plant leaves is not yet established, plastochromanol-8 accumulation could participate in the phototolerance of the SPS1-overexpressing plants. However, this idea is not consistent with the phenotype of VTE1oex transgenic plants which accumulated large amounts of plastochromanol-8 with normal plastoquinone-9 levels and close to normal tocopherol levels: the latter plants exhibited the same tolerance towards photooxidative stress as WT (Fig. 8). Thus, high plastochromanol-8 levels do not provide obvious advantages to leaves, at least under the light stress conditions used in our study, indicating that accumulation of plastochromanols cannot explain by itself the increased phototolerance of the SPS1oex plants.

Interestingly, concomitant suppression of plastochromanol-8 and tocopherols in the *vte1* mutant had a strong impact on the plastoquinone-9 levels which were substantially lowered compared to the WT levels both in low light and in high light (Fig. 7). Such an effect was not observed in the tocopherol-deficient *vte2* mutant that contains normal levels of plastochromanol-8. Moreover, the decreased pool of plastoquinone-9 in the *vte1* mutant was accompanied by a much higher sensitivity to high light stress compared to *vte2*. This difference between the *vte1* and *vte2* mutants can be understood if we consider that prenyl lipids can have overlapping antioxidant functions. In the absence of both tocopherols and plastochromanol-8, photoprotection provided by the latter compounds relies now on plastoquinone-9. Due to the enhanced solicitation of plastoquinone-9 as an antioxidant, its concentration drops as it is consumed during ^1^O_2_ scavenging. Cooperation between antioxidants in protection against photosensitized oxidation is known for carotenoids and tocopherols^59^; a similar phenomenon could occur between prenyl lipids.

A substantial fraction of the prenyl lipids plastoquinone-9, plastochromanol-8 and tocopherols is stored in the plastoglobules that are attached to the thylakoid membranes^35^,^47^,^60^. We found that, under the growth conditions used in this study, about 50% of plastoquinones are present in the plastoglobules of WT Arabidopsis leaves (Fig. 5). The plastoquinone molecules contained in the plastoglobules are not directly connected to the photosynthetic electron transport chain and therefore they represent the photochemically non-active fraction of plastoquinone. This non-photoactive pool was shown to represent the majority of plastoquinones in high light-grown Arabidopsis plants^34^. In several other plant species, increased concentrations of plastoquinone-9 (*e.g*. with aging or high PFDs) correlate with appearance of plastoglobules^47^. The same phenomenon appeared to occur in the SPS1oex plants in which the extra plastoquinones were mostly non-photoactive. Plastoglobules are permanently coupled to thylakoid membranes, indicating that prenyl lipids have the possibility to diffuse from plastoglobules to thylakoids^61^. Actually, the rapid consumption of about 80% of the plastoquinones observed in the present study in Arabidopsis plants suddenly exposed to excess light energy (Fig. 1B) supports the idea of plastoquinone exchange between plastoglobules and thylakoids. Since the majority of plastoquinone-9 is located in the plastoglobules, high light-induced degradation of such a high percentage of the plastoquinone content implies that plastoquinone molecules moved from the plastoglobules to the thylakoid-based photosystems where ROS are produced and can interact with them. In this context, it is also interesting to note that knockdown of *FIBBRILIN4* gene expression in apple disrupted the partitioning of plastoquinone between the plastoglobules and the rest of the chloroplast at the expenses of the plastoglobules^62^ and increased concomitantly the sensitivity to biotic and abiotic stresses including high light^63^. Plastoglobular accumulation of plastoquinone-9 and rapid channeling of the stored plastoquinone-9 molecules to the thylakoids could thus function as a photoprotective mechanism by maintaining efficient ^1^O_2_-scavenging activity under high light illumination and by providing new plastoquinone molecules for the PSII repair cycle. Stimulation of plastoquinone synthesis in SPS1oex plants is likely to enhance this mechanism, thus leading to more phototolerant plants as observed in this study.

In conclusion, it is becoming clear that the function of plastoquinones goes beyond their traditional roles as photosynthetic electron carriers between PSII and PSI and redox signals regulating cellular activities^20^,^21^. In fact, the partitioning of plastoquinone-9 between thylakoid membranes and their attached plastoglobules underlies the involvement of this compound in different physiological mechanisms. It has been recently shown that the plastoquinones contained in the plastoglobules are involved in electron-transfer reactions important for the chloroplast metabolism^36^. Other possible functions have recently emerged from a number of *in vitro* studies that have revealed that plastoquinone-9 possesses antioxidant properties^23^,^24^. Our finding that SPS1 overexpression in Arabidopsis concomitantly boosts the plastoquinone biosynthesis pathway and the plant tolerance to photooxidative stress demonstrates that this class of molecules does fulfill a protective role *in planta*.

## Methods

### Plant material

WT Arabidopsis plants (*Arabidopsis thaliana*, ecotype Col 0 or Col 2) were grown at an air temperature of 20 °C/18 °C (day/night), a PFD of ~170 μmol photons m^−2^ s^−1^ with a photoperiod of 8 h (unless specified otherwise) and a relative air humidity of 70%. The following mutants were used in this study: the tocopherol cyclase mutant *vte1*^38^, the homogentisate phytyl transferase mutant *vte2*^39^, the ^1^O_2_-producing mutant *ch1*^28^, and two VTE1 overexpressing lines, one in the *vte1* mutant background (VTE1oex #1) and the other in the WT background (VTE1oex #40)^40^. The corresponding WT (Col 0 or Col 2) of the different mutants are indicated in the figure legends.

Experiments were done on plants aged 4 or 5 weeks, as specified in the figure legends. Biochemical/biophysical analyses were performed on mature, well-developed leaves. High light stress was imposed by transferring plants to a high PFD (1300 μmol photons m^−2^ s^−1^) at a low air temperature of 5 or 8 °C. Under those stress conditions, leaf temperature was ca. 17 or 19 °C respectively, in the temperature range of unstressed leaves. Because of its high photosensitivity^28^, the *ch1* mutant plants were submitted to less severe stress conditions (1000 μmol photons m^−2^ s^−1^ at 10 °C).

### Prenyl lipids

Leaf discs were grinded in ethyl acetate. After centrifugation, the surpernatant was filtered, evaporated on ice under a stream of N_2_, recovered in methanol/hexane (17/1) and filtered before analysis by HPLC, as described in^64^ and^34^. The column was a Phenomenex Kinetex 2.6 μm, 100 × 4.6 mm, 100 A. Separation of tocopherols, plastoquinone-9 and plastochromanol-8 was done in the isocratic mode with methanol/hexane (17/1) as solvent system and a flow rate of 0.8 ml/min. All prenyl lipids, except oxidized plastoquinone-9, were detected by their fluorescence at 330 nm with an excitation at 290 nm. Plastoquinone-9 in the oxidized state was measured by its absorbance at 255 nm. Plastochromanol-8 and plastoquinone-9 standards were a kind gift from Dr. J. Kruk. Tocopherol standards were purchased from Sigma.

Leaf samples were frozen in liquid nitrogen as quickly as possible to avoid changes in the plastoquinone redox state during sample preparation. Determination of the photoactive and non-active fractions of plastoquinone-9 was done following the protocol described in^64^. Leaves were cut in half. To determine the pool of photoactive plastoquinone-9, we first measured the amount of reduced plastoquinone-9 (plastoquinol-9) in one half of the leaves after a dark-adaptation period of 2 h. The dark-adapted samples were frozen in liquid nitrogen after 3-s illumination with far-red light to ensure full oxidation of the photoactive pool of plastoquinone. Then, plastoquinol-9 was re-quantified in the other half of the leaves that had been exposed to a high PFD of white light (2000 μmol photons m^−2^ s^−1^) for 15 s. Illumination was done in a mortar that was filled with liquid nitrogen after the 15-s illumination in order to freeze the samples in the light. The size of the photoactive pool was calculated as the difference between the plastoquinol-9 content in the light and the plastoquinol-9 content in the dark. The size of the non-photoactive pool of plastoquinone-9 was the sum of the amount of plastoquinol-9 in the dark (not reoxidable in the dark) and the amount of oxidized plastoquinone-9 in the light (not reducible by high light). This pool corresponds mainly to the plastoquinone molecules present in the plastoglobules although a small fraction can also be present in the chloroplast envelope^47^.

### Chlorophyll fluorescence

Chlorophyll fluorescence was measured in attached leaves with a PAM-2000 chlorophyll fluorometer (Walz), as described in^15^. The maximal quantum yield of PSII photochemistry was measured after a period of dark adaptation as Fv/Fm = (Fm-Fo)/Fm where Fo is the initial fluorescence level (measured with a weak, non-actinic red light) and Fm is the maximal fluorescence level (measured with a 800-ms pulse of saturating white light). The actual quantum yield of PSII photochemistry was measured in the light as ΔF/Fm’ = (Fm’-Fs)/Fm where Fs is the steady-state fluorescence level and Fm’ is the maximal fluorescence level. ΔF/Fm’ was measured in leaves adapted to different PFDs of white light.

### RNA Isolation and Quantitative RT-PCR

The NucleoSpin RNA plant kit (Macherey-Nagel) was used to extract total RNA from 100 mg of leaves. RNA concentrations were measured on a NanoDrop2000 (Thermo Scientific, USA). Quantitative RT-PCR (qRT-PCR) was performed with cDNA synthesized with the Prime Script^TM^ Reverse Transcriptase (Takara) from 500 ng of total RNA. Design of the specific primers was done for each gene (see supplemental Table 1) with the Primer3plus software (www.bioinformatics.nl/cgi-bin/primer3plus/primer3plus.cgi). qRT-PCR was carried out using the LightCycler 480 SYBR Green I Master (Roche) in the quantitative PCR thermal cycler (LightCycler 480 real-time PCR system; Roche). Reaction preparation (total volume of 5 μl): 2 μl of cDNA diluted 10-fold, 2 μl of SYBR Green I Master, and 1 μM forward and reverse primers. The amplification profile was 95 °C for 10 min and 45 cycles (95 °C/15 s, 60 °C or 56 °C (depending on the gene)/15 s, and 72 °C/15 s). Reactions were performed in triplicates. Normalization of gene expressions was done using *ACTIN2* as housekeeping gene. At least three biological replicates were performed for each gene tested.

### SPS1 overexpressing transgenic Arabidopsis plants

To generate plants over-expressing *SPS1* (At1g78510), full-length cDNA was amplified using the primers allowing the addition of attB recombination sites. The cDNA was cloned into a pDONR201 vector (Life Technologies) and transferred into the binary GATEWAY destination vector pB2GW7,0 (Plant Systems Biology, VIB-Ghent University, Belgium)^65^. The pB2GW7,0 vector allows expression of a sense cDNA under the control of the cauliflower mosaic virus 35S promoter and includes a glufosinate-resistance gene. Transformation using *Agrobacterium tumefaciens* C58 strain was performed as described by Davis *et al*.^66^. Homozygous transgenic lines (T3) were produced and selected from resistance segregation assays.

Transformed lines were verified using PCR analysis on genomic DNA with the Phire Plant Direct PCR kit (Finnzymes) and gene-specific primers. ACTIN2 was used as a positive control for each PCR. The PCR program was 98 °C for 5 min, 30 cycles (denaturation at 95 °C for 5 s, annealing at 55 °C for 5 s, extension at 72 °C for 20 s) (GeneAmp, PCRSystem 2700, Applied Biosystems). For RT-PCR analysis, RNA extraction from leaves was done using NucleoSpin RNA Plant Kit (Macherey Nagel) according to the manufacturer’s instructions. Reverse transcription was performed using total RNA (500 ng), a Superscript III Reverse Transcriptase (Life Technologies) and an oligo(dT)20 primer. RT-PCR assays were performed using the same gene-specific primers used for genotyping. Primers: *SPS1*-F, tggacgcctgctttatctct; *SPS1*-R, gcaagcttgatacacgacga; *ACTIN2*-F, aaaatggccgatggtgaggatat; *ACTIN2*-R, caataccggttgtacgaccact.

### Lipid peroxidation analysis and imaging

0.5 g plant material (fresh weight) was grinded with Ultraturax T25 (Ika-Werk) in 2.5 ml CHCl_3_-methanol (50-50) containing 5mM triphenyl phosphine, 1mM butylated hydroxytoluene and 1 ml citric acid (1M), with 15-hydroxy-11,13(*Z,E*)-eicosadienoic acid as internal standard. After evaporation under a stream of N_2_, the organic phase was re-solubilized in 1.25 ml ethanol and 1.25 ml 3.5 M NaOH. The sample was hydrolyzed at 80 °C for 30 min. pH was adjusted between 4 and 5 by addition of 1 M citric acid, and hydroxy fatty acids were then extracted with hexane/ether (50/50). HOTE isomers (hydroxy octadecatrienoic acid, produced by the oxidation of linolenic acid) were separated and quantified by straight phase HPLC-UV analysis, as described in a previous work^67^. Estimation of ROS-induced HOTEs and lipoxygenase-induced HOTEs was done according to the method described in^67^.

Lipid peroxidation was imaged by measuring autoluminescence emission. The latter signal is attributed to the spontaneous decomposition of lipid peroxides^68^. Spontaneous photon emission from whole plants was measured after a 2-h dark adaptation period using a cooled charge-coupled device (CCD) camera, as explained in^68^. Integration time was 20 min and pixel binning was 2 × 2. The images were analyzed with the ImageJ software.

## Additional Information

**How to cite this article**: Ksas, B. *et al*. Plant tolerance to excess light energy and photooxidative damage relies on plastoquinone biosynthesis. *Sci. Rep*. **5**, 10919; doi: 10.1038/srep10919 (2015).

## Supplementary Material

Supplementary Information
