# Supplementary material for: Plant tolerance to excess light energy and photooxidative damage relies on plastoquinone biosynthesis
Source: Sci Rep. 2015 Jun 3;5:10919. doi: 10.1038/srep10919 (PMC4454199; doi:10.1038/srep10919)
Supplement: Supplementary Information [file srep10919-s1.pdf]

Plant tolerance to excess light energy and photooxidative damage relies on plastoquinone biosynthesis

Brigitte Ksas, Noëlle Becuwe, Anne Chevalier & Michel Havaux

Supplemental Table 1. List of genes and oligonucleotide primer sequences used for the gene expression analysis by qRT-PCR.

Name	Locus	Forward primer seq. (5'-3')	Reverse primer seq. (5'-3')	Length (pb)
**SPS1**	At1g78510	GCAAGCTTGATACACGACGA	TGGACGCCTGCTTTATCTCT	**220**
**SPS2**	At1g17050	TGATTTGCAGAGGTTGAACG	ATCTCACCTAAACGCCGATG	**198**
**HST**	At3g11950	TGCTGCAATGGTGAAAGAAG	CTTCTGGAGCTTTGGTTTCG	**179**
**HPPD**	At1g06570	CGACTTCATGCCTTCTCCTC	AAATATCGTCGGCCTGTCAC	**181**
**VTE1**	At4g32770	ACTAGTTCCCCGCTCCATTT	CTCGAAGAACTTCCGAGGTG	**169**
**VTE3**	At3g63410	CCAAAGCAAAGCAAAAGGAG	GCTTCCCTTATTCCCCTCTG	**151**
**ACTIN2**	At3g18780	CTCTCCCGCTATGTATGTCGC	GAAACCCTCGTAGATTGGCA	124

Percentage reduction state of plastoquinone-9 in WT and *ch1* leaves

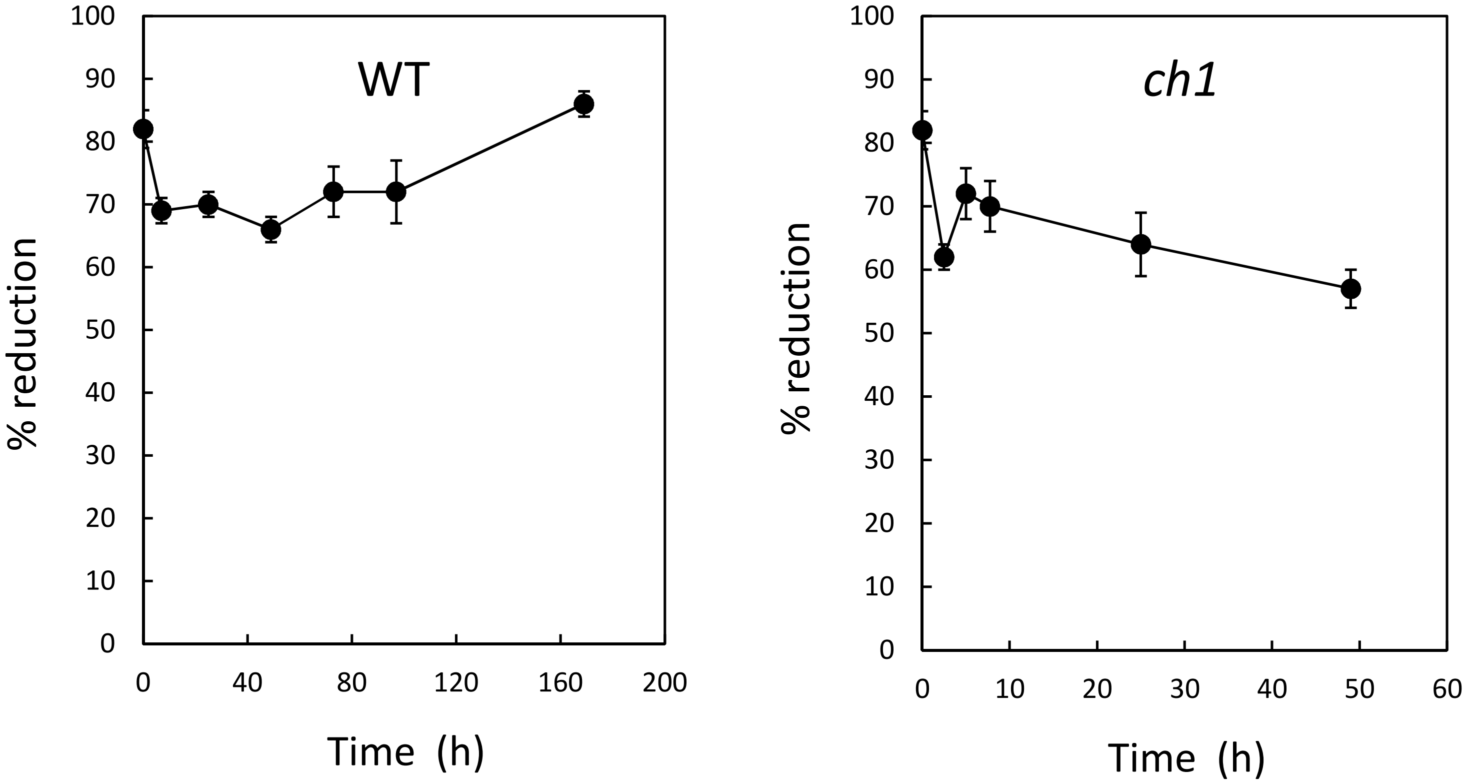

Supplementary Fig. 1: Percentage reduction state of plastoquinone-9 (ratio between reduced plastoquinone-9 and total plastoquinone x100) in WT and *ch1* Arabidopsis leaves during high light stress (1300 µmol m^{-2} s^{-1} at 8°C).

Percentage reduction state of plastoquinone-9 in WT and SPS1oex leaves

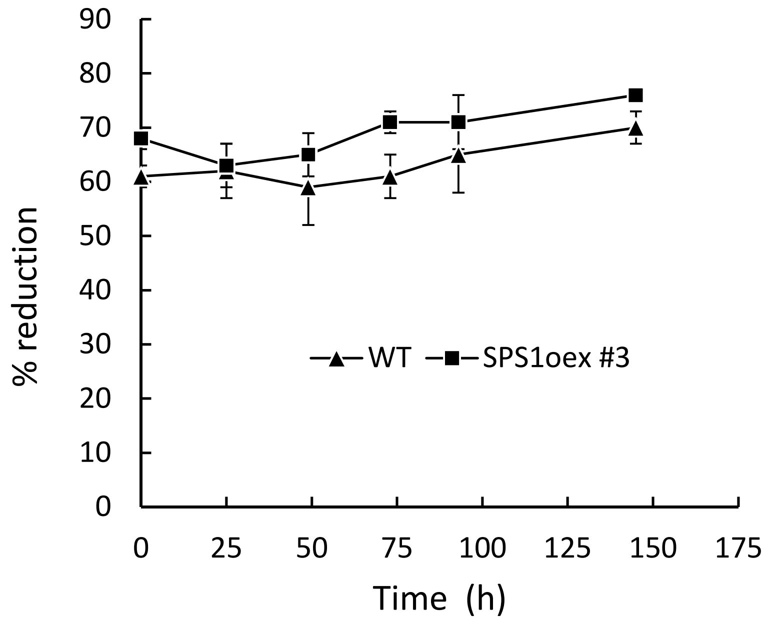

Supplementary Fig. 2: Percentage reduction state of plastoquinone-9 in WT and SPS1oex leaves during high light stress (1300 μmol m^{-2} s^{-1} at 8°C).

Growth and photosynthesis of WT and SPS1oex plants

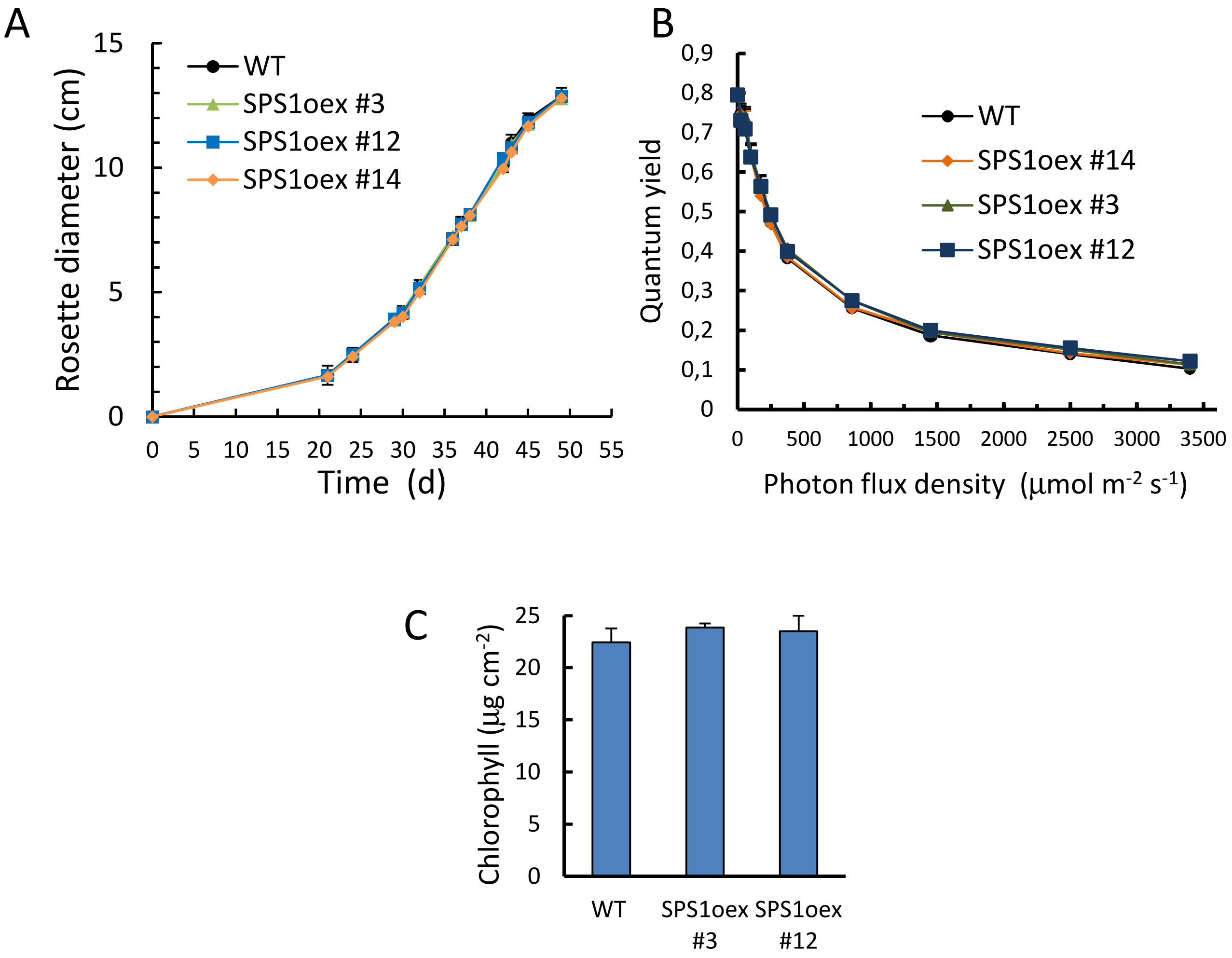

Supplementary Fig. 3: A) Growth curve of WT Arabidopsis and 3 SPS1oex lines. Data are mean value of 6 measurements ± SD. B) Quantum yield of photosynthetic electron transport (ΔF/Fm') at different PFDs. Data are mean values of 5 separate experiments + SD. C) Total chlorophyll. Data are mean values of 4 separate experiments + SD.
